# Dietician-based approach to lipodystrophic syndrome: role of bioelectrical impedance analysis at week 12

**DOI:** 10.1186/1758-2652-13-S4-P77

**Published:** 2010-11-08

**Authors:** G Orofino, M Guastavigna, P Martinoglio, D Penoncelli, D Demarie, M Tettoni, P Desiderato, R Di Frenna, C Zignin

**Affiliations:** 1 ASL TO 2, Ambulatorio lipodistrofie e dismetabolismi, Torino, Italy

## Background

Lipodystrophic syndrome (LS) is the expression of HIV infection chronicity and negatively affect survival, adherence and quality of life. About 30% of patients suffer from LS; among them, 30% need a specific treatment. We developed a multidisciplinary diagnostic and therapeutic program funded by Regione Piemonte. The patient is guided step by step, aimed at working on changeable lifestyles, to reverse metabolic alterations and to cope with the distress related to change in body image.

## Methods

The project is carried out in two regional hospitals in Piemonte. The team is formed by specialists in HIV disease, a nutritionist, a dietician, a psychologist, a cardiologist, a plastic surgeon and a kinesiologist. The first step is the metabolic assessment, then the psychological visit and the dietician follow-up. It is based on clinical and educational aspects of dietetic therapy (i.e. dietetic diary and nutritional counseling) and it starts with Bioelectrical Impedance Analysis (BIA). We use the Tanita BC 418 MA® Segmental Body Composition Analyser that prints out a body profile including weight, body fat percentage, body fat mass (BFM), fat free mass, estimated muscle mass and basal metabolic rate. Dietician visits are at time 0,1,3,6,12 months. The patients are also invited to participate in a psychological homogeneous group and in a group of physical activity (Nordic Walking).

## Results

At date 99 patients have been enrolled: 40 females, 59 males; mean age 48,9 years; mean age of HIV infection 9,9 years; risk factors for HIV infection: 20 IVUD, 79 sexual intercourse; CDC Classification: 47 A, 27 B, 25 C. At baseline 39 (39%) patients had also metabolic syndrome; 36 (36%) had cardiovascular risk at 10 years ≥10%; 66 (66%) had BMI≥ 25. At date, 10 (10%) patients dropped out after the metabolic assessment; 11 (11%) underwent a cardiological visit. 38 patients were assessed with BIA at baseline and at week 12: 26/38 (69%) significantly decreased their BMI and their BFM and 8/26 (31%) improve plasma lipids, especially triglycerides (TG) (2 of them started pharmacological therapy to decrease TG); 10/38 (26%) had no significant BMI and BFM variation; 2/38 (5%) increased BMI and BFM.

## Conclusions

The dietician intervention together with a simple, reliable, repeatable and low cost examination can establish a pattern to observe and improve important metabolic parameters. The follow-up will clarify if early BIA variations are predictive markers of long-term outcome.

